# Routes to embodiment

**DOI:** 10.3389/fpsyg.2015.00940

**Published:** 2015-07-02

**Authors:** Anita Körner, Sascha Topolinski, Fritz Strack

**Affiliations:** ^1^Department of Psychology II, University of Würzburg, Würzburg, Germany; ^2^Social and Economic Cognition, Department of Psychology, University of Cologne, Cologne, Germany

**Keywords:** embodied cognition, grounded cognition, simulation, priming, metaphors

## Abstract

Research on embodiment is rich in impressive demonstrations but somewhat poor in comprehensive explanations. Although some moderators and driving mechanisms have been identified, a comprehensive conceptual account of how bodily states or dynamics influence behavior is still missing. Here, we attempt to integrate current knowledge by describing three basic psychological mechanisms: *direct state induction*, which influences how humans feel or process information, unmediated by any other cognitive mechanism; *modal priming*, which changes the accessibility of concepts associated with a bodily state; *sensorimotor simulation*, which affects the ease with which congruent and incongruent actions are performed. We argue that the joint impact of these mechanisms can account for most existing embodiment effects. Additionally, we summarize empirical tests for distinguishing these mechanisms and suggest a guideline for future research about the mechanisms underlying embodiment effects.

## Introduction

Carrying weight makes hills seem steeper and distances seem greater (Bhalla and Proffitt, 1999; Proffitt et al., 2003). Additionally it influences judgments about observed weight lifting in others (Hamilton et al., 2004); but also estimates of a topic’s importance (Jostmann et al., 2009; Chandler et al., 2012), of one’s guilt (Kouchaki et al., 2014), and of one’s success in learning (Alban and Kelley, 2013). These findings highlight the body’s role in information processing, termed embodiment. Conceptually, however, this impressively diverse set of effects is poorly integrated (Schubert and Semin, 2009; Barsalou, 2010; Meier et al., 2012; Willems and Francken, 2012; Glenberg et al., 2013).

Recently, some moderators and driving mechanisms of specific embodiment effects have been identified. For example, interoceptive sensitivity was found to moderate different embodiment effects (Häfner, 2013), and fishy smells’ impact on distrust was linked to a chain of semantic associations (Lee and Schwarz, 2012). However, a comprehensive conceptual account of how bodily states or dynamics influence human experience and behavior is still missing. Therefore, it is our goal to integrate current knowledge by describing three basic psychological mechanisms and illustrate them briefly with findings from the cognitive, social psychology, and neuroscience literature (for more extensive reviews, see, e.g., Barsalou, 2008; Landau et al., 2010; Glenberg et al., 2013).

To start out, we adopt a phenomenological definition for embodiment. Specifically, we define embodiment as an effect where the body, its sensorimotor state, its morphology, or its mental representation play an instrumental role in information processing. This definition is agnostic about how such knowledge is represented (for more theory-driven accounts of how to define embodiment, see, e.g., Wilson, 2002; Goldman and de Vignemont, 2009). Even though the question about how knowledge is grounded, and to what degree this grounding is based on the body, is a vital one, our classification of embodiment effects does not explicitly make use of knowledge representations. Therefore, we leave the question of knowledge representation to theories of embodied cognition. Accordingly, some of the effects we discuss do not provide evidence for stronger versions of embodied or grounded cognition hypotheses (e.g., Clark, 1999; Chemero, 2013; see also Wilson, 2002; Machery, 2007) but can also be explained by amodal mechanisms (i.e., mechanisms that are completely independent of modality-specific systems). Indeed, we also discuss amodal mechanisms for embodiment effects. Our primary goal is to compare, and classify the effects and understand their underlying mechanisms. Hopefully, this will advance our understanding of the body’s role in information processing and shed light on the more fundamental mechanisms of cognition and the nature of the cognitive architecture.

Pursuing this goal, we suggest a classification for embodiment effects that is based on their underlying mechanisms. Specifically, we argue that sensations or actions can have three qualitatively different effects on the mind.

• First, they can directly alter a person’s state of mind, feelings, or information processing (*direct state induction*).
• Second, they can change how readily specific information comes to mind, thus influencing the mental contents instead of the mode of operation (*modal priming*).
• Third, they can lead to compatibility effects with concurrent automatic simulations, changing, for example, fluency and preferences (*sensorimotor simulation*).

We classify all three mechanisms as automatic in the sense that they require little processing resources and can be initiated unintentionally (see Moors and De Houwer, 2006). However, this does not mean these mechanisms are uncontrollable or inflexible. On the contrary, we adopt a situated cognition perspective (Smith and Semin, 2004); thus, especially modal priming and sensorimotor simulation are generally influenced, among other things, by a person’s current goal state and by situational circumstances.

In the following, we sketch each route to embodiment and highlight its signature characteristics. We do not go into details about the mechanisms, as all of them have been proposed before and explained more fully than could be done here^1^. Instead, we highlight and compare the mechanisms and their features. Our key thesis is that each mechanism has distinct features that cannot easily be explained by any of the other mechanisms. This enables us to provide procedures to test their respective contributions for a given empirical effect.

## Mechanism 1: Direct State Induction

Some bodily states directly induce affective or non-affective feelings and are not mediated by any kind of higher cognitive mechanism such as attributions or inferences (Barsalou et al., 2003; Neumann et al., 2003; Niedenthal, 2007). For example, keeping muscle tension associated with increasing or decreasing distance may induce a motivational orientation of avoidance or approach, respectively (Cacioppo et al., 1993; Strack and Deutsch, 2004). Similarly, disabling the corrugator muscle (by an injection of Botulinum Toxin A) may reduce negative affect and depressive symptoms (Lewis and Bowler, 2009; Wollmer et al., 2012). Thus, with facial configuration directly influencing emotional experiences, direct state induction is akin to William James’s theory of emotion elicitation (James, 1884) and to some later theories of emotion, like facial feedback theory and hard interface theory (Zajonc and Markus, 1988). Accordingly, this route to embodiment should generally be stable, in terms of both context influences and temporal dynamics.

The resulting psychological state can, in turn, also influence judgment or behavior. For example, distance changing muscle contractions were found to influence diverse judgments and behaviors, such as valence judgments (Cacioppo et al., 1993; Centerbar and Clore, 2006), the recognition of valenced information (Förster and Strack, 1996), the amount of consumed food (Förster, 2003), tendencies to judge others as similar or dissimilar to oneself (Nussinson et al., 2010), and the recruitment of different cognitive resources (Friedman and Förster, 2005; Koch et al., 2008).

Direct state induction is similar to procedural or mindset priming. Both are content free (Gollwitzer, 1990; Förster et al., 2009), which implies that the activation refers not to distinct concepts but to the information processing style, influencing cognitive functions in unrelated domains. Moreover, it does not matter how the altered state or mindset came about. The same motivational state may influence cognitive processes in the same manner, regardless if the state was elicited by a cognitive or an embodied induction. For instance, an avoidance orientation should result in a tendency to evaluate neutral stimuli as negative and to adopt a narrow attentional scope—no matter if it was induced by walking backward (Fayant et al., 2011), by arm extension (Cacioppo et al., 1993), or by performing a task of guiding a mouse through a maze to avoid a bird of prey (Friedman and Förster, 2005).

Even though direct state induction works largely independently of contextual and situational factors, the elicited state may be prevented from influencing behavior, for instance by re-attributing the feeling (Schwarz, 2011). Thus, like physical arousal’s influence on a judgment can be moderated by attributing it to an incidental source (e.g., a pill) instead of one’s reaction to the stimulus (Schwarz et al., 1985), the influence of bodily induced feelings on behavior can be counteracted. Therefore, finding high context sensitivity for an embodiment manipulation does not rule out direct state induction as the responsible mechanism. Instead, it is crucial to distinguish between the induced state and the resulting behavior. While the former is assumed to follow directly from the embodiment manipulation, the latter is highly flexible and context sensitive. Thus, direct state induction alters a person’s state or mindset. But, how this state and mindset is used depends on a number of factors besides the state itself.

## Mechanism 2: Modal Priming

In modal priming, sensorimotor states activate (often abstract) concepts. For example, handling a rough (vs. smooth) object leads to judging social interactions to be less coordinated (Ackerman et al., 2010). Similarly, holding a heavy (vs. light) object heightens the perceived significance of an object or the weight of a topic (Jostmann et al., 2009; Schneider et al., 2011). This mechanism presupposes that representations contain modality-specific aspects (e.g., movements or bodily, facial, or sensory states). Activating the bodily states partially activates the associated semantic concepts (e.g., by means of spreading activation in a multi-modal associative store; see Smith and DeCoster, 2000; Strack and Deutsch, 2004; for a connectionist model for a modal priming effect, see Flusberg et al., 2010); and, in turn, the activated semantic concepts can influence behavior.

Modal priming effects have gained much attention through reports of surprising associations. Instead of being semantically associated, the bodily states or actions and the abstract concepts are often connected via conceptual metaphors (Lakoff and Johnson, 1980, 1999; Landau et al., 2010). For example, a faint smell of fish decreases trust in social interactions (Lee and Schwarz, 2012). Yet, the connection between fish and trust consists solely in the metaphoric expression of “something smelling fishy” for untrustworthiness.

Apart from its distinct mode of activation, modal priming operates similar to priming in general (Lee and Schwarz, 2012; Meier et al., 2012). Specifically, the bodily manipulation increases the accessibility of associated concepts making them more likely to be used in subsequent tasks (Loersch and Payne, 2011). Importantly, accessible concepts can be used to either construct a target or a standard of comparison (Schwarz and Bless, 1992; Bless and Schwarz, 2010), leading to assimilation or contrast effects, respectively. Also, they can be discarded altogether when they are attributed to a source that is irrelevant for the task at hand (see also Wheeler et al., 2007, for a similar account).

Like other kinds of priming, modal priming effects should in general be bidirectional. Thus, while a fishy smell activates the concept of suspiciousness, inducing suspicion also lowers the sensory threshold to detect fishy smells (Lee and Schwarz, 2012). Similarly, walking slowly activates the elderly concept (Mussweiler, 2006), while, conversely, activating the elderly concept decreases walking speed (Bargh et al., 1996). Although there is convincing evidence for the unidirectionality of some effects (e.g., Boroditsky, 2000; Casasanto and Boroditsky, 2008), the majority of metaphoric effects have been shown to work bidirectionally. Similarly, for priming in general, bidirectionality is the rule rather than the exception.

In fact, a similar analogy holds for *modal* and *semantic priming* to the previously described analogy of *direct state induction* and *mindset priming*. As long as a concept is activated and attributed to one’s own judgment about a currently perceived stimulus, similar effects result whether the concept is activated semantically or physically (IJzerman and Semin, 2010; see also DeWall and Bushman, 2009; Dimmock et al., 2013). From this analogy follow some properties of *modal priming* and *direct state induction*: *modal priming* influences specific associated concepts while *direct state induction* affects a broader range of behaviors. Moreover, the time course should differ. While content that is activated through priming gets quickly deactivated, altered information processing tends to last longer after the manipulation (Smith and Branscombe, 1987).

## Mechanism 3: Sensorimotor Simulation

Perceiving a stimulus automatically triggers the simulation of reenacting or interacting with it (Barsalou, 1999, 2008). For instance, seeing objects that afford handling evokes the simulation of grasping (Tucker and Ellis, 1998), reading words elicits the simulation of pronunciation (Fadiga et al., 2002; Topolinski and Strack, 2009) and reading sentences leads to a multi-sensory simulation of the experiential content (Stanfield and Zwaan, 2001; Glenberg and Kaschak, 2002; Pecher et al., 2003; Fischer and Zwaan, 2008). This automatic simulation is very similar to the action or sensation itself—even employing the same brain regions (Gallese, 2007). Taking this notion farther, mental representation might essentially be “the reenactment of previous experiences” (Pecher and Winkielman, 2013, p. 396).

As a result, sensations or actions are facilitated if they are congruent with the simulated sensations or actions, while incompatible sensations or actions are hampered. For example, pressing the right (or left) key in response to an image of a cup with its handle on the right (or left) side is facilitated compared to the mismatching assignment (Tucker and Ellis, 1998).

Simulation is important in social interactions. Understanding actions in others involves activity in brain regions involved in performing these very actions. Indeed, a network of brain regions (sometimes referred to as mirror-neuron system) is activated both when an action is performed and when it is observed in others (Buccino et al., 2001; Rizzolatti and Craighero, 2004; cf. Prinz, 1990). Evidence for a causal role of those brain regions in action understanding comes from lesion and interference studies (e.g., Urgesi et al., 2007; Pazzaglia et al., 2008). For example, temporarily impairing participants’ hand (vs. lip) area of the premotor cortex (by repetitive transcranial magnetic stimulation) increases error rates in understanding pantomimed hand (vs. lip) actions (Michael et al., 2014). Thus, in addition to its quality of reenacting previous experiences, simulation has an additional function in predicting other’s behavior (Gallese, 2007, 2009; see also Borghi and Cimatti, 2010).

Simulation has also been shown to play a causal role in various other mental faculties, such as processing emotion (Oberman et al., 2007; Foroni and Semin, 2009, 2011, 2012; Niedenthal et al., 2009) or representing meaning (Klatzky et al., 1989; Zwaan and Taylor, 2006)—even the meaning of abstract concepts (e.g., information transfer, Glenberg and Kaschak, 2002). Moreover, preference and esthetic appreciation also relies on sensorimotor simulation, specifically its fluency (Beilock and Holt, 2007; Topolinski and Strack, 2009).

Automatic simulation depends on previous experience and skills (Beilock et al., 2008). Accordingly, participants trained in a specific movement are better than untrained participants at visually recognizing similar movements (Casile and Giese, 2006). Similarly, participants with severe spinal-cord injury are impaired in detecting biological motion (Arrighi et al., 2011). Moreover, young children learn about other people’s goal-directed object manipulations by interacting with objects themselves (Sommerville et al., 2005; see also Campos et al., 2000).

A unique feature of the simulation mechanism is that it also works offline, that is, in absence of the particular bodily state or action that is simulated (Wilson, 2002; see also Niedenthal et al., 2005). A stimulus is processed and elicits a simulation even without any particular sensorimotor state; this simulation—its ease or the associated sensorimotor activity—affects information processing, judgment, and behavior. From this property it follows that a bodily manipulation can be used to both facilitate and interfere with the simulation (e.g., Beilock and Holt, 2007; Elder and Krishna, 2012).

Moreover, simulation is more flexible in how and when it takes place than the other two mechanisms. Simulation occurs automatic, unintentionally, fast, and even without attending to the stimulus eliciting the simulation (Shtyrov et al., 2014). However, it can be blocked by a concurrent task using the same sensorimotor resources (e.g., chewing blocks covert pronunciation, Topolinski and Strack, 2009) and can even be prevented by performing some tasks on the stimuli (Solomon and Barsalou, 2004; Sato et al., 2008; Niedenthal et al., 2009; Papeo et al., 2009; for an overview, see also Willems and Casasanto, 2011). As yet, it is not entirely clear which conditions prevent simulation, and whether these conditions are independent of simulation contents (e.g., emotions or actions), but it seems that simulation is a default mechanism that occurs unless it is prevented.

## Mixed Forms

We do not argue that every embodiment effect is exclusively driven by one of these three routes. Instead, joint influences are rather the rule than the exception. For instance, there seem to be two driving mechanisms for embodied cleansing. Embodied cleansing refers to the effect that physical cleansing one’s hands reduces the impact of previous experiences (e.g., washing one’s hands after a transgression reduces guilty feelings, Zhong and Liljenquist, 2006). On the one hand, several studies found semantic associations between (im)morality and (un)cleanness. Specifically, activating the concept of immorality leads to higher accessibility of cleaning-related words (Zhong and Liljenquist, 2006; Jones and Fitness, 2008; Yan et al., 2011), which points to modal priming. On the other hand, cleaning influences a broad range of cognitive processes in domains beyond morality, called the “clean slate effect” (Lee and Schwarz, 2010; Xu et al., 2012); and diversity of consequences is a signature feature of direct state induction. A similar case can be made for the influence of weight on importance judgments. Here, both modal priming (Zhang and Li, 2012) and sensorimotor simulation (Häfner, 2013) have been argued to contribute to the effect.

## The Role of Conscious Inferences

Inferential processes may also play a role in combination with the three automatic mechanisms discussed thus far. Specifically, the bodily state or action can be consciously perceived and categorized, such that inferences can be drawn from it, which may contribute to the observed effect. One such inferential process could be self-perception. Typically, *self-perception* has been ruled out in embodiment by the use of unobtrusive bodily manipulations and elaborate debriefings.

However, inferential processes can also influence the *accessibility* of concepts. A conscious classification of an action that is inherently ambiguous can be necessary for the activation of a concept, and thus for the occurrence of modal priming. For example, clothes make the man—depending on man’s interpretation of the clothes: Wearing a white coat increases participants’ performance in attention-related tasks when the coat is introduced as a doctor’s coat compared to a painter’s coat (Adam and Galinsky, 2012). This is another instance of a mixed effect as both the conscious label and actual wearing of the coat are necessary for the effect to occur (Adam and Galinsky, 2012; see also Boroditsky and Ramscar, 2002; Casasanto and Dijkstra, 2010, for related notions about the influence of a movement’s subjective meaning).

Conscious inferences can also influence or even reverse compatibility effects of sensorimotor simulation. In general, changing distance leads to a compatibility effect with the valence of a stimulus (i.e., moving toward a positive stimulus and away from a negative one are faster than *vice versa*; e.g., Solarz, 1960; Chen and Bargh, 1999). However, giving a different meaning to the movement can reverse the effect (Markman and Brendl, 2005; Eder and Rothermund, 2008; Seibt et al., 2008). Interestingly, when combining re-categorization of the movement and automatic tendencies to increase or decrease distance, both influence movement speed depending on the valence of the word (Krieglmeyer et al., 2010). So it seems that both automatic distance regulation and the situational meaning of a movement produce compatibility effects between a word’s valence and movement.

## Disentangling the Routes to Embodiment

The current topology of three routes to embodiment does not only parsimoniously cover the bulk of existing embodiment effects with a few basic principles, it also provides empirical tests of their respective contributions to a given phenomenon. Let us illustrate this with the well-known pen manipulation (Strack et al., 1988), where participants hold a pencil either between their protruded lips with their teeth (while not touching it with the lips) or with their lips. This procedure was meant to either facilitate or inhibit the contraction of the zygomaticus muscle which is used in smiling. At the same time, participants were prevented from categorizing their facial action as a smile and draw inferences from it. While the smile facilitation condition yielded higher funniness ratings of cartoons compared to a neutral control condition, inhibited smiling caused lower funniness ratings (Strack et al., 1988).

The pen manipulation could reasonably be based on all three proposed mechanisms. First, operating through direct state induction, smiling might improve mood. Yet, when assessing participants’ affective state, generally no influence of the pen manipulation can be detected (Strack et al., 1988; Niedenthal et al., 2001). Second, operating through modal priming, smiling could activate fun-related concepts which could in turn be used as judgmental cues in the funniness ratings. However, this is also unlikely because holding a pen between one’s teeth does not lead to faster responses for positively valenced words in a lexical decision task (Havas et al., 2007). Third and most likely, automatic simulations of subtle smiling could be triggered when evaluating funniness (Foroni and Semin, 2009). These smiling simulations could be influenced by concurrent facilitated or inhibited smiling as a result of holding a pen. And indeed, facilitated or inhibited sensorimotor simulations seem to lead to respective effects on evaluations (Havas and Matheson, 2013). Moreover, only sensorimotor simulation explains the decrease in funniness ratings by an inhibition of smiling compared to a no-interference control condition. Thus, the facial posture’s influence on funniness ratings seems to involve sensorimotor simulation.

In the following we outline tests for each causal route, exploiting its respective procedural features such as breadth of effects or arbitrariness of associations.

### Probing Direct State Induction

Direct state induction requires the activation of a global psychological state, emotion, or mindset, which allows for the following tests.

#### Presence of Induced State

As its name says, the test of direct state induction is assessing the presence of the induced state. Recurring to the smile facilitation example, smiling could improve positive mood which could lead to cartoons being judged funnier. But as the effect on cartoon judgment has been repeatedly observed without concurrent mood change, this mechanism is unlikely. Of course, assessing the focal state depends on the measure’s sensitivity—for instance, smile induction might elicit affect too subtle to be caught by mood reports—and agreement on what the state might be. If, however, the proposed state does vary with the manipulation, and if this variation mediates the effect of the manipulation on the dependent measure, then direct state induction seems responsible for producing the embodiment effect at hand.

#### Diversity of Consequences

Usually, states induced by embodiment are global psychological states, such as emotions or motivational orientations, prompting a broad variety of cognitive, affective, and behavioral consequences. Thus, the scope of impact should usually be broader for direct state induction than for the other routes—particularly concerning consequences in content domains that are not connected to the bodily manipulation. For instance, the link between arm muscle contraction and performance in the Stroop task (Koch et al., 2008) cannot be reconstructed by semantic associations or sensorimotor simulation.

#### Universal State Induction

As direct state induction does not rest on stored semantic or linguistic associations, inducing a certain sensorimotor state should invariably result in a similar state change—across languages, cultures, and other factors that shape semantic memory. Thus, cross-cultural replications of the same effect speak in favor of direct state induction. In contrast, particularly modal priming, often involving culturally idiosyncratic language metaphors, should substantially be modulated by culture (see the next section).

### Probing Modal Priming

Modal priming generally conforms to the rules of priming more generally, which allows for the following tests.

#### Activation of Concepts Instead of Global States

In modal priming, embodiment manipulations invariably activate associated concepts. Indeed, this concept activation is thought to mediate any effects. Therefore, the most direct test is to measure concept accessibility, for instance, via a lexical decision or word stem completion task (Lee and Schwarz, 2012). If the concepts supposedly mediating an embodiment effect are not activated by the manipulation, modal priming is an unlikely mechanism—as in the case of no heightened accessibility of positive valence words by the pen manipulation (Havas et al., 2007). In contrast to the rather broad affective, cognitive, and behavioral consequences following direct state induction (see the preceding section), concept activation should be relatively more narrow and specific and always traceable through associative links.

#### Dependence on Associative Structures

As any other priming form, modal priming depends on the architecture of the associative network and thus on cultural, linguistic, biographic, and many other factors shaping this associative structure. Therefore, for example, cultural practices, linguistic metaphors, expertise, and interindividual differences should influence the presence and direction of modal priming effects. This is, for instance, the case for the mental representation of time which varies with linguistic metaphors in a culture (Boroditsky, 2001; Boroditsky and Gaby, 2010).

#### Arbitrariness and Flexibility of Associations

One unique feature of modal associations is that they can be arbitrary. Although for many associations an ecological connection seems plausible, this is not necessary. Arbitrary cultural conventions, for instance, showing hostility by extending one’s middle finger (Chandler and Schwarz, 2009), can form strong associations via learning and thus yield embodiment effects. Furthermore, these associations can be transformed via learning, so that experimental conditioning of associations should alter embodiment effects that rest upon modal priming. In contrast, the bodily conditions in direct state inductions are rather phylogenetically shaped and rigid states; and their relations to psychological effects are hard-wired and less flexible, such as emotions and their affective and cognitive syndromes (Russell, 2003).

### Probing Sensorimotor Simulation

Simulation takes place automatically, in the absence of the sensation or action that is simulated, which allows for the following tests.

#### Interference

The unique feature of sensorimotor simulation is its susceptibility to interference and complete blockade (e.g., Beilock and Holt, 2007; Foroni and Semin, 2009; Topolinski and Strack, 2009). Thus, to test simulation, a concurrent task that engages the same sensorimotor resources as the simulation should interfere with the simulation. If an effect relies partly on simulation, the effect should be diminished under such interventions. For example, participants with blocked frowning muscles are slower to read negative emotional sentences (Havas et al., 2010).

Note that a facilitated simulation effect is not as strong support for simulation as an inhibited simulation effect. Both effects would be expected when simulation contributes to an effect, but facilitation could also be explained by modal priming while inhibition could not. Having all three conditions in the same experiment (i.e., facilitation, inhibition, and a neutral condition, where simulation is neither facilitated nor inhibited) constitutes a strong test for simulation. Even more so, as alternative explanations (e.g., distraction) are ruled out if the manipulations for interference and facilitation are similar.

#### Fluency

Another—and, where feasible, particularly neat—test for simulation consists in altering the fluency of the simulation. Participants trained in a specific movement are better than control participants at simulating that movement, and consequently profit at related tasks (Casile and Giese, 2006; Topolinski, 2010; Leder et al., 2013). Additionally, simulation fluency can be altered by “untraining” a more fluent action. For example, people generally prefer objects according to their handedness—right-handers prefer objects on their right side, and left-handers on their left side (Casasanto, 2009; Elder and Krishna, 2012; Shen and Sengupta, 2012). However, performing movements where the dominant hand is made relatively clumsy reverses this fluency, which in turn reverses preferences (Casasanto and Chrysikou, 2011). This kind of temporarily altering fluency by training has the advantage that no manipulation has to be present when the effect is measured. Moreover, for disfluency training, priming accounts would rather make the opposite prediction from simulation. The training increases frequency of prior concept activation and thereby should lead to an increase in accessibility, while sensorimotor simulation predicts a decreased embodiment effect as a result of disfluency training.

### Further Tests

#### Conditions for Contrast Effects

Up to now, most published embodiment effects show an assimilative pattern. However, in sensorimotor simulation, specific differences in timing (Reed and McGoldrick, 2007) or similarity between current and simulated sensorimotor state (Jacobs and Shiffrar, 2005) can change facilitation into interference and can thereby lead to contrast effects (see also Zwickel and Prinz, 2012). In modal priming, though up to now not empirically tested, experimental manipulations that influence whether the activated knowledge is used to construct the target or the standard of comparison might play a greater role in reversing assimilation into contrast effects (see the mechanism description). Third, in direct state induction, contrast effects should never occur in the induced state—unless brought about by additionally working inferential processes.

#### Bidirectionality

Turning the independent variable in embodiment studies, the bodily state, into the dependent variable and testing whether the converse effect holds as well, has sometimes been suggested as a test for sensorimotor simulation against modal priming (e.g., Schneider et al., 2011). However, as outlined above, most modal priming effects are bidirectional—as are effects driven by direct state induction and sensorimotor simulation. Thus, bidirectionality is no distinguishing characteristic for the presently proposed mechanisms.

## Conclusion

We have proposed three mechanisms of how the human body, its actions and states, can influence human cognition and behavior—each with distinct properties and distinct consequences. Crucially, we argue that across psychological subdisciplines, these mechanisms and their joint impact can account for the bulk of existing embodiment effects^2^.

On the other hand, classifying embodiment effects with respect to subject matter or semantic category seems less suitable when causal mechanisms are of interest, because effects within the same subject domain may be caused by different mechanisms. For example, approach–avoidance effects can be differentially caused, either because they are directly induced by a motivational orientation or because they are (in)compatible with a sensorimotor simulation. Thus, for embodiment to become a truly useful integrative framework for psychology (Schubert and Semin, 2009; Glenberg, 2010), it is necessary to understand its underlying mechanisms. We believe our framework is a valuable contribution that can be tested and refined in the future.

Additionally, we have derived empirical standards for how the working of each of these mechanisms can be tested for a given effect, and how the specific contributions of a joint impact of these routes can be dissociated. With that we aim to provide a guideline for future research targeted at understanding the operating mechanisms. A systematically spelled-out distinction of underlying processes with tests to distinguish the processes is vital when trying to resolve debates about what might cause a certain embodiment effect. For example, the current controversy about the effects of expanded posture on power or power-related states or behavior (see Huang et al., 2011; Cesario and McDonald, 2013; Park et al., 2013) could profit from clear statements about what is considered the basic state affected by an expanded posture, and what constitutes decisive tests for distinguishing the mechanism.

Recent theoretical work has advanced distinctions of embodiment effects similar to ours. Specifically our first two mechanisms somewhat resemble Cohen and Leung’s (2009) pre-wired (direct state induction) and totem (modal priming) embodiments, respectively. Both pre-wired embodiment and direct state induction are seen as stable and universal. Similarly, both totem embodiments and modal priming rely heavily on learning. However, direct state induction is a narrower category than pre-wired embodiment, while modal priming is broader and can include non-arbitrary associations. Moreover, our conceptualization seems to be more comprehensive, encompassing both purely cognitive and sensorimotor simulation effects. Moreover, by focusing on the mechanisms, our framework is naturally more explicit about cognitive processes.

Other research programs are more distantly related to our approach. For example, simulation effects can be split into online and offline effects (Niedenthal et al., 2005). This distinction can help elucidating the many different effects that simulation can have on cognition and behavior, but essentially rests on one mechanism. Other approaches investigate the phylogenetic and ontogenetic precursors of embodiment (e.g., Williams et al., 2009; Bargh et al., 2012; Casasanto, 2014). While certainly an intriguing question, it does not necessarily advance our knowledge of the underlying mechanisms, as there is no one-to-one mapping of the developmental course and operating mechanism. Similarly, investigating where the boundaries of embodiment are, which functions are embodied, and to what degree (e.g., Mahon and Caramazza, 2008; Louwerse and Jeuniaux, 2010), will certainly advance our understanding of information processing, but it is an endeavor quite distinct from distinguishing different embodiment mechanisms when they are operating.

To conclude, we have proposed a comprehensive framework consisting of three distinct mechanisms that explain how the body may shape the mind. With this distinction and our emphasis on empirical tests for the proposed mechanisms, we hope to stimulate further research on the mechanisms that underlie the surprising phenomena of embodiment.

### Conflict of Interest Statement

The authors declare that the research was conducted in the absence of any commercial or financial relationships that could be construed as a potential conflict of interest.

## Footnotes

1 Indeed, the mechanisms have even been proposed in this or similar form to explain the effects we use as illustrations for the mechanisms, though sometimes without any empirical support for the working of the proposed mechanism.

2 One mechanism that we did not mention relies on polarity correspondence (Proctor and Cho, 2006; Lakens, 2012). That is, some congruency effects between physical and psychological dimensions can be explained convincingly solely by polarity differences instead of invoking priming or simulation.

## References

[B1] Ackerman J. Nocera C. Bargh J. A. (2010). Incidental haptic sensations influence social judgments and decisions. Science 328, 1712–1715. 10.1126/science.1189993 20576894 PMC3005631

[B2] Adam H. Galinsky A. D. (2012). Enclothed cognition. J. Exp. Soc. Psychol. 48, 918–925. 10.1016/j.jesp.2012.02.008

[B3] Alban M. W. Kelley C. M. (2013). Embodiment meets metamemory: weight as a cue for metacognitive judgments. J. Exp. Psychol. Learn. Mem. Cogn. 39, 1628–1634. 10.1037/a0032420 23565793

[B4] Arrighi R. Cartocci G. Burr D. (2011). Reduced perceptual sensitivity for biological motion in paraplegia patients. Curr. Biol. 21, R910–R911. 10.1016/j.cub.2011.09.048 22115454

[B5] Bargh J. Chen M. Burrows L. (1996). Automaticity of social behavior: direct effects of trait construct and stereotype activation on action. J. Pers. Soc. Psychol. 71, 230–244. 10.1037/0022-3514.71.2.230 8765481

[B6] Bargh J. A. Schwader K. L. Hailey S. E. Dyer R. L. Boothby E. J. (2012). Automaticity in social-cognitive processes. Trends Cogn. Sci. 16, 593–605. 10.1016/j.tics.2012.10.002 23127330

[B7] Barsalou L. (1999). Perceptual symbol systems. Behav. Brain Sci. 22, 577–660. 10.1017/S0140525X99002149 11301525

[B8] Barsalou L. (2008). Grounded cognition. Annu. Rev. Psychol. 59, 617–645. 10.1146/annurev.psych.59.103006.093639 17705682

[B9] Barsalou L. (2010). Grounded cognition: past, present, and future. Top. Cogn. Sci. 2, 716–724. 10.1111/j.1756-8765.2010.01115.x 25164052

[B10] Barsalou L. Niedenthal P. Barbey A. Ruppert J. (2003). “Social embodiment,” in Psychology of Learning and Motivation, Vol. 43, ed. Ross B. (San Diego, CA: Academic Press), 43–92.

[B11] Beilock S. Holt L. (2007). Embodied preference judgments. Psychol. Sci. 18, 51–57. 10.1111/j.1467-9280.2007.01848.x 17362378

[B12] Beilock S. Lyons I. Mattarella-Micke A. Nusbaum H. Small S. (2008). Sports experience changes the neural processing of action language. Proc. Natl. Acad. Sci. U.S.A. 105, 13269–13273. 10.1073/pnas.0803424105 18765806 PMC2527992

[B13] Bhalla M. Proffitt D. R. (1999). Visual–motor recalibration in geographical slant perception. J. Exp. Psychol. Hum. Percept. Perform. 25, 1076–1096. 10.1037/0096-1523.25.4.1076 10464946

[B14] Bless H. Schwarz N. (2010). Mental construal and the emergence of assimilation and contrast effects: the inclusion/exclusion model. Adv. Exp. Soc. Psychol. 42, 319–373. 10.1016/S0065-2601(10)42006-7

[B15] Borghi A. M. Cimatti F. (2010). Embodied cognition and beyond: acting and sensing the body. Neuropsychologia 48, 763–773. 10.1016/j.neuropsychologia.2009.10.029 19913041

[B16] Boroditsky L. (2000). Metaphoric structuring: understanding time through spatial metaphors. Cognition 75, 1–28. 10.1016/S0010-0277(99)00073-6 10815775

[B17] Boroditsky L. (2001). Does language shape thought? Mandarin and English speakers’ conceptions of time. Cogn. Psychol. 43, 1–22. 10.1006/cogp.2001.0748 11487292

[B18] Boroditsky L. Gaby A. (2010). Remembrances of times east: absolute spatial representations of time in an Australian aboriginal community. Psychol. Sci. 21, 1635–1639. 10.1177/0956797610386621 20959511

[B19] Boroditsky L. Ramscar M. (2002). The roles of body and mind in abstract thought. Psychol. Sci. 13, 185–189. 10.1111/1467-9280.00434 11934006

[B20] Buccino G. Binkofski F. Fink G. R. Fadiga L. Fogassi L. Gallese V. (2001). Action observation activates premotor and parietal areas in a somatotopic manner: an fMRI study. Eur. J. Neurosci. 13, 400–404. 10.1111/j.1460-9568.2001.01385.x 11168545

[B21] Cacioppo J. T. Priester J. R. Berntson G. G. (1993). Rudimentary determinants of attitudes: II. Arm flexion and extension have differential effects on attitudes. J. Pers. Soc. Psychol. 65, 5–17. 10.1037/0022-3514.65.1.5 8355142

[B22] Campos J. J. Anderson D. I. Barbu-Roth M. A. Hubbard E. M. Hertenstein M. J. Witherington D. (2000). Travel broadens the mind. Infancy 1, 149–219. 10.1207/S15327078IN0102_1 32680291

[B23] Casasanto D. (2009). Embodiment of abstract concepts: good and bad in right- and left-handers. J. Exp. Psychol. Gen. 138, 351–367. 10.1037/a0015854 19653795

[B24] Casasanto D. (2014). “Experiential origins of mental metaphors: Language, culture, and body,” in The Power of Metaphor: Examining its Influence on Social Life, eds Landau M. J. Robinson M. D. Meier B. (Washington, DC: American Psychological Association), 249–268. 10.1037/14278-011

[B25] Casasanto D. Boroditsky L. (2008). Time in the mind: using space to think about time. Cognition 106, 579–593. 10.1016/j.cognition.2007.03.004 17509553

[B26] Casasanto D. Chrysikou E. (2011). When left is “right”: motor fluency shapes abstract concepts. Psychol. Sci. 22, 419–422. 10.1177/0956797611401755 21389336

[B27] Casasanto D. Dijkstra K. (2010). Motor action and emotional memory. Cognition 115, 179–185. 10.1016/j.cognition.2009.11.002 20096831 PMC2830276

[B28] Casile A. Giese M. A. (2006). Nonvisual motor training influences biological motion perception. Curr. Biol. 16, 69–74. 10.1016/j.cub.2005.10.071 16401424

[B29] Centerbar D. B. Clore G. L. (2006). Do approach-avoidance actions create attitudes? Psychol. Sci. 17, 22–29. 10.1111/j.1467-9280.2005.01660.x 16371140

[B30] Cesario J. McDonald M. M. (2013). Bodies in context: power poses as a computation of action possibility. Soc. Cogn. 31, 260–274. 10.1521/soco.2013.31.2.260

[B31] Chandler J. Reinhard D. Schwarz N. (2012). To judge a book by its weight you need to know its content: knowledge moderates the use of embodied cues. J. Exp. Soc. Psychol. 48, 948–952. 10.1016/j.jesp.2012.03.003

[B32] Chandler J. Schwarz N. (2009). How extending your middle finger affects your perception of others: learned movements influence concept accessibility. J. Exp. Soc. Psychol. 45, 123–128. 10.1016/j.jesp.2008.06.012

[B33] Chemero A. (2013). Radical embodied cognitive science. Rev. Gen. Psychol. 17, 145–150. 10.1037/a0032923

[B34] Chen M. Bargh J. A. (1999). Consequences of automatic evaluation: immediate behavioral predispositions to approach or avoid the stimulus. Pers. Soc. Psychol. Bull. 25, 215–224. 10.1177/0146167299025002007

[B35] Clark A. (1999). An embodied cognitive science? Trends Cogn. Sci. 3, 345–351. 10.1016/s1364-6613(99)01361-3 10461197

[B36] Cohen D. Leung A. (2009). The hard embodiment of culture. Eur. J. Soc. Psychol. 39, 1278–1289. 10.1002/ejsp.671

[B37] DeWall C. N. Bushman B. J. (2009). Hot under the collar in a lukewarm environment: words associated with hot temperature increase aggressive thoughts and hostile perceptions. J. Exp. Soc. Psychol. 45, 1045–1047. 10.1016/j.jesp.2009.05.003

[B38] Dimmock J. A. Jackson B. Clarke D. (2013). “Warm interactions” and “Cold streaks”: the influence of temperature word primes on interpersonal warmth and competence-related outcomes in a dyadic sport task. Sport Exerc. Perform. Psychol. 2, 102–116. 10.1037/a0030191

[B39] Eder A. B. Rothermund K. (2008). When do motor behaviors (mis)match affective stimuli? An evaluative coding view of approach and avoidance reactions. J. Exp. Psychol. Gen. 137, 262–281. 10.1037/0096-3445.137.2.262 18473659

[B40] Elder R. S. Krishna A. (2012). The “visual depiction effect” in advertising: facilitating embodied mental simulation through product orientation. J. Consum. Res. 38, 988–1003. 10.1086/661531

[B41] Fadiga L. Craighero L. Buccino G. Rizzolatti G. (2002). Speech listening specifically modulates the excitability of tongue muscles: a TMS study. Eur. J. Neurosci. 15, 399–402. 10.1046/j.0953-816x.2001.01874.x 11849307

[B42] Fayant M.-P. Muller D. Nurra C. Alexopoulos T. Palluel-Germain R. (2011). Moving forward is not only a metaphor: approach and avoidance lead to self-evaluative assimilation and contrast. J. Exp. Soc. Psychol. 47, 241–245. 10.1016/j.jesp.2010.07.013

[B43] Fischer M. H. Zwaan R. A. (2008). Embodied language: a review of the role of the motor system in language comprehension. Q. J. Exp. Psychol. 61, 825–850. 10.1080/17470210701623605 18470815

[B44] Flusberg S. J. Thibodeau P. H. Sternberg D. A. Glick J. J. (2010). A connectionist approach to embodied conceptual metaphor. Front. Psychol. 1:197. 10.3389/fpsyg.2010.00197 21833256 PMC3153806

[B45] Foroni F. Semin G. (2009). Language that puts you in touch with your bodily feelings. The multimodal responsiveness of affective expressions. Psychol. Sci. 20, 974–980. 10.1111/j.1467-9280.2009.02400.x 19594858

[B46] Foroni F. Semin G. R. (2011). When does mimicry affect evaluative judgment? Emotion 11, 687–690. 10.1037/a0023163 21668115

[B47] Foroni F. Semin G. R. (2012). Not all implicit measures of attitudes are created equal: evidence from an embodiment perspective. J. Exp. Soc. Psychol. 48, 424–427. 10.1016/j.jesp.2011.08.015

[B48] Förster J. (2003). The influence of approach and avoidance motor actions on food intake. Eur. J. Soc. Psychol. 33, 339–350. 10.1002/ejsp.150

[B49] Förster J. Liberman N. Friedman R. S. (2009). “What do we prime? On distinguishing between semantic priming, procedural priming, and goal priming,” in Oxford Handbook of Human Action. Social Cognition and Social Neuroscience, eds Morsella E. Bargh J. A. Gollwitzer P. M. (New York, NY: Oxford University Press), 173–193.

[B50] Förster J. Strack F. (1996). Influence of overt head movements on memory for valenced words: a case of conceptual-motor compatibility. J. Pers. Soc. Psychol. 71, 421–430. 10.1037/0022-3514.71.3.421 8831156

[B51] Friedman R. S. Förster J. (2005). The influence of approach and avoidance cues on attentional flexibility. Motiv. Emot. 29, 69–81. 10.1007/s11031-005-7954-4

[B52] Gallese V. (2007). Before and below ‘Theory of Mind’: embodied simulation and the neural correlates of social cognition. Philos. Trans. R. Soc. B Biol. Sci. 362, 659–669. 10.1098/rstb.2006.2002 17301027 PMC2346524

[B53] Gallese V. (2009). Motor abstraction: a neuroscientific account of how action goals and intentions are mapped and understood. Psychol. Res. 73, 486–498. 10.1007/s00426-009-0232-4 19381683

[B54] Glenberg A. M. (2010). Embodiment as a unifying perspective for psychology. Wiley Interdiscip. Rev. Cogn. Sci. 1, 586–596. 10.1002/wcs.55 26271505

[B55] Glenberg A. M. Kaschak M. (2002). Grounding language in action. Psychon. Bull. Rev. 9, 558–565. 10.3758/BF03196313 12412897

[B56] Glenberg A. M. Witt J. K. Metcalfe J. (2013). From the revolution to embodiment: 25 years of cognitive psychology. Perspect. Psychol. Sci. 8, 573–585. 10.1177/1745691613498098 26173215

[B57] Goldman A. I. de Vignemont F. (2009). Is social cognition embodied? Trends Cogn. Sci. 13, 154–159. 10.1016/j.tics.2009.01.007 19269881

[B58] Gollwitzer P. M. (1990). “Action phases and mind-sets,” in Handbook of Motivation and Cognition: Foundations of Social Behavior, eds Higgins E. T. Sorrentino R. M. (New York, NY: Guilford Press), 53–92.

[B59] Hamilton A. Wolpert D. Frith U. (2004). Your own action influences how you perceive another person’s action. Curr. Biol. 14, 493–498. 10.1016/j.cub.2004.03.007 15043814

[B60] Havas D. A. Glenberg A. Gutowski K. Lucarelli M. Davidson R. (2010). Cosmetic use of botulinum toxin-A affects processing of emotional language. Psychol. Sci. 21, 895–900. 10.1177/0956797610374742 20548056 PMC3070188

[B61] Havas D. A. Glenberg A. Rinck M. (2007). Emotion simulation during language comprehension. Psychon. Bull. Rev. 14, 436–441. 10.3758/BF03194085 17874584

[B62] Havas D. A. Matheson J. (2013). The functional role of the periphery in emotional language comprehension. Front. Psychol. 4:294. 10.3389/fpsyg.2013.00294 23750145 PMC3664318

[B63] Häfner M. (2013). When body and mind are talking: interoception moderates embodied cognition. Exp. Psychol. 60, 255–259. 10.1027/1618-3169/a000194 23548985

[B64] Huang L. Galinsky A. D. Gruenfeld D. H. Guillory L. E. (2011). Powerful postures versus powerful roles: which is the proximate correlate of thought and behavior? Psychol. Sci. 22, 95–102. 10.1177/0956797610391912 21149853

[B65] IJzerman H. Semin G. R. (2010). Temperature perceptions as a ground for social proximity. J. Exp. Soc. Psychol. 46, 867–873. 10.1016/j.jesp.2010.07.015

[B66] Jacobs A. Shiffrar M. (2005). Walking perception by walking observers. J. Exp. Psychol. Hum. Percept. Perform. 31, 157–169. 10.1037/0096-1523.31.1.157 15709870

[B67] James W. (1884). What is an emotion? Mind 9, 188–205. 10.1093/mind/os-IX.34.188

[B68] Jones A. Fitness J. (2008). Moral hypervigilance: the influence of disgust sensitivity in the moral domain. Emotion 8, 613–627. 10.1037/a0013435 18837611

[B69] Jostmann N. Lakens D. Schubert T. (2009). Weight as an embodiment of importance. Psychol. Sci. 20, 1169–1174. 10.1111/j.1467-9280.2009.02426.x 19686292

[B70] Klatzky R. L. Pellegrino J. W. McCloskey B. P. Doherty S. (1989). Can you squeeze a tomato? The role of motor representations in semantic sensibility judgments. J. Mem. Lang. 28, 56–77. 10.1016/0749-596X(89)90028-4

[B71] Koch S. Holland R. W. van Knippenberg A. (2008). Regulating cognitive control through approach-avoidance motor actions. Cognition 109, 133–142. 10.1016/j.cognition.2008.07.014 18835601

[B72] Kouchaki M. Gino F. Jami A. (2014). The burden of guilt: heavy backpacks, light snacks, and enhanced morality. J. Exp. Psychol. Gen. 143, 414–424. 10.1037/a0031769 23398182

[B73] Krieglmeyer R. Deutsch R. De Houwer J. De Raedt R. (2010). Being moved valence activates approach-avoidance behavior independently of evaluation and approach-avoidance intentions. Psychol. Sci. 21, 607–613. 10.1177/0956797610365131 20424109

[B74] Lakens D. (2012). Polarity correspondence in metaphor congruency effects: structural overlap predicts categorization times for bipolar concepts presented in vertical space. J. Exp. Psychol. Learn. Mem. Cogn. 38, 726–736. 10.1037/a0024955 21843022

[B75] Lakoff G. Johnson M. (1980). Metaphors We Live By. Chicago, IL: University of Chicago press.

[B76] Lakoff G. Johnson M. (1999). Philosophy in the Flesh: The Embodied Mind and its Challenge to Western Thought. New York, NY: Basic books.

[B77] Landau M. J. Meier B. P. Keefer L. A. (2010). A metaphor-enriched social cognition. Psychol. Bull. 136, 1045–1067. 10.1037/a0020970 20822208

[B78] Leder H. Bär S. Topolinski S. (2013). Covert painting simulations influence aesthetic appreciation of artworks. Psychol. Sci. 23, 1479–1481. 10.1177/0956797612452866 23137968

[B79] Lee S. W. S. Schwarz N. (2010). Washing away postdecisional dissonance. Science 328, 709. 10.1126/science.1186799 20448177

[B80] Lee S. W. S. Schwarz N. (2012). Bidirectionality, mediation, and moderation of metaphorical effects: the embodiment of social suspicion and fishy smells. J. Pers. Soc. Psychol. 103, 737–749. 10.1037/a0029708 22905770

[B81] Lewis M. B. Bowler P. J. (2009). Botulinum toxin cosmetic therapy correlates with a more positive mood. J. Cosmet. Dermatol. 8, 24–26. 10.1111/j.1473-2165.2009.00419.x 19250162

[B82] Loersch C. Payne B. K. (2011). The situated inference model: an integrative account of the effects of primes on perception, behavior, and motivation. Perspect. Psychol. Sci. 6, 234–252. 10.1177/1745691611406921 26168515

[B83] Louwerse M. M. Jeuniaux P. (2010). The linguistic and embodied nature of conceptual processing. Cognition 114, 96–104. 10.1016/j.cognition.2009.09.002 19818435

[B84] Machery E. (2007). Concept empiricism: a methodological critique. Cognition 104, 19–46. 10.1016/j.cognition.2006.05.002 16814274

[B85] Mahon B. Caramazza A. (2008). A critical look at the embodied cognition hypothesis and a new proposal for grounding conceptual content. J. Physiol. Paris 102, 59–70. 10.1016/j.jphysparis.2008.03.004 18448316

[B86] Markman A. B. Brendl C. M. (2005). Constraining theories of embodied cognition. Psychol. Sci. 16, 6–10. 10.1111/j.0956-7976.2005.00772.x 15660844

[B87] Meier B. P. Schnall S. Schwarz N. Bargh J. A. (2012). Embodiment in social psychology. Top. Cogn. Sci. 4, 705–716. 10.1111/j.1756-8765.2012.01212.x 22777820

[B88] Michael J. Sandberg K. Skewes J. Wolf T. Blicher J. Overgaard M. (2014). Continuous theta-burst stimulation demonstrates a causal role of premotor homunculus in action understanding. Psychol. Sci. 25, 963–972. 10.1177/0956797613520608 24549297 PMC4232279

[B89] Moors A. De Houwer J. (2006). Automaticity: a theoretical and conceptual analysis. Psychol. Bull. 132, 297–326. 10.1037/0033-2909.132.2.297 16536645

[B90] Mussweiler T. (2006). Doing is for thinking! Stereotype activation by stereotypic movements. Psychol. Sci. 17, 17–21. 10.1111/j.1467-9280.2005.01659.x 16371139

[B91] Neumann R. Förster J. Strack F. (2003). “Motor compatibility: the bidirectional link between behavior and evaluation,” in The Psychology of Evaluation: Affective Processes in Cognition and Emotion, eds Musch J. Klauer K. C. (Mahwah, NJ: Lawrence Erlbaum Associates), 371–391.

[B92] Niedenthal P. (2007). Embodying emotion. Science 316, 1002–1005. 10.1126/science.1136930 17510358

[B93] Niedenthal P. Barsalou L. Winkielman P. Krauth-Gruber S. Ric F. (2005). Embodiment in attitudes, social perception, and emotion. Pers. Soc. Psychol. Rev. 9, 184–211. 10.1207/s15327957pspr0903_1 16083360

[B94] Niedenthal P. Brauer M. Halberstadt J. Innes-Ker Å. (2001). When did her smile drop? Facial mimicry and the influences of emotional state on the detection of change in emotional expression. Cogn. Emot. 15, 853–864. 10.1080/02699930143000194

[B95] Niedenthal P. Winkielman P. Mondillon L. Vermeulen N. (2009). Embodiment of emotion concepts. J. Pers. Soc. Psychol. 96, 1120–1136. 10.1037/a0015574 19469591

[B96] Nussinson R. Seibt B. Häfner M. Strack F. (2010). Come a bit closer: approach motor actions lead to feeling similar and behavioral assimilation. Soc. Cogn. 28, 40–58. 10.1521/soco.2010.28.1.40

[B97] Oberman L. Winkielman P. Ramachandran V. (2007). Face to face: blocking facial mimicry can selectively impair recognition of emotional expressions. Soc. Neurosci. 2, 167–178. 10.1080/17470910701391943 18633815

[B98] Papeo L. Vallesi A. Isaja A. Rumiati R. I. (2009). Effects of TMS on different stages of motor and non-motor verb processing in the primary motor cortex. PLoS ONE 4:e4508. 10.1371/journal.pone.0004508 19240793 PMC2643000

[B99] Park L. E. Streamer L. Huang L. Galinsky A. D. (2013). Stand tall, but don’t put your feet up: universal and culturally-specific effects of expansive postures on power. J. Exp. Soc. Psychol. 49, 965–971. 10.1016/j.jesp.2013.06.001

[B100] Pazzaglia M. Pizzamiglio L. Pes E. Aglioti S. M. (2008). The sound of actions in apraxia. Curr. Biol. 18, 1766–1772. 10.1016/j.cub.2008.09.061 19013068

[B101] Pecher D. Winkielman P. (2013). “Grounded cognition and social interaction,” in Encyclopedia of Philosophy and the Social Sciences, ed. Kaldis B. (Thousand Oaks, CA: Sage), 396–397.

[B102] Pecher D. Zeelenberg R. Barsalou L. W. (2003). Verifying different-modality properties for concepts produces switching costs. Psychol. Sci. 14, 119–124. 10.1111/1467-9280.t01-1-01429 12661672

[B103] Prinz W. (1990). “A common-coding approach to perception and action,” in Relationships Between Perception and Action: Current Approaches, eds Neumann O. Prinz W. (Berlin: Springer), 167–201.

[B104] Proctor R. W. Cho Y. S. (2006). Polarity correspondence: a general principle for performance of speeded binary classification tasks. Psychol. Bull. 132, 416–442. 10.1037/0033-2909.132.3.416 16719568

[B105] Proffitt D. R. Stefanucci J. Banton T. Epstein W. (2003). The role of effort in perceiving distance. Psychol. Sci. 14, 106–112. 10.1111/1467-9280.t01-1-01427 12661670

[B106] Reed C. L. McGoldrick J. E. (2007). Action during body perception: processing time affects self–other correspondences. Soc. Neurosci. 2, 134–147. 10.1080/17470910701376811 18633812

[B107] Rizzolatti G. Craighero L. (2004). The mirror-neuron system. Annu. Rev. Neurosci. 27, 169–192. 10.1146/annurev.neuro.27.070203.144230 15217330

[B108] Russell J. A. (2003). Core affect and the psychological construction of emotion. Psychol. Rev. 110, 145–172. 10.1037/0033-295X.110.1.145 12529060

[B109] Sato M. Mengarelli M. Riggio L. Gallese V. Buccino G. (2008). Task related modulation of the motor system during language processing. Brain Lang. 105, 83–90. 10.1016/j.bandl.2007.10.001 18054379

[B110] Schneider I. Rutjens B. Jostmann N. Lakens D. (2011). Weighty matters: importance literally feels heavy. Soc. Psychol. Pers. Sci. 2, 474–478. 10.1177/1948550610397895

[B111] Schubert T. Semin G. (2009). Embodiment as a unifying perspective for psychology. Eur. J. Soc. Psychol. 39, 1135–1141. 10.1002/ejsp.670

[B112] Schwarz N. (2011). “Feelings-as-information theory,” in Handbook of Theories of Social Psychology, eds Van Lange P. Kruglanski A. W. Higgins E. T. (Thousand Oaks, CA: Sage), 289–308.

[B113] Schwarz N. Bless H. (1992). “Constructing reality and its alternatives: an inclusion/exclusion model of assimilation and contrast effects in social judgment,” in The Construction of Social Judgments, eds Martin L. L. Tesser A. (Hillsdale, NJ: Lawrence Erlbaum Associates), 217–245.

[B114] Schwarz N. Servay W. Kumpf M. (1985). Attribution of arousal as a mediator of the effectiveness of fear-arousing communications. J. Appl. Soc. Psychol. 15, 74–78. 10.1111/j.1559-1816.1985.tb02343.x

[B115] Seibt B. Neumann R. Nussinson R. Strack F. (2008). Movement direction or change in distance? Self-and object-related approach–avoidance motions. J. Exp. Soc. Psychol. 44, 713–720. 10.1016/j.jesp.2007.04.013

[B116] Shen H. Sengupta J. (2012). If you can’t grab it, it won’t grab you: the effect of restricting the dominant hand on target evaluations. J. Exp. Soc. Psychol. 48, 525–529. 10.1016/j.jesp.2011.11.003

[B117] Shtyrov Y. Butorina A. Nikolaeva A. Stroganova T. (2014). Automatic ultrarapid activation and inhibition of cortical motor systems in spoken word comprehension. Proc. Natl. Acad. Sci. U.S.A. 111, E1918–E1923. 10.1073/pnas.1323158111 24753617 PMC4020094

[B118] Smith E. R. Branscombe N. R. (1987). Procedurally mediated social inferences: the case of category accessibility effects. J. Exp. Soc. Psychol. 23, 361–382. 10.1016/0022-1031(87)90036-9

[B119] Smith E. R. DeCoster J. (2000). Dual-process models in social and cognitive psychology: conceptual integration and links to underlying memory systems. Pers. Soc. Psychol. Rev. 4, 108–131. 10.1207/S15327957PSPR0402_01

[B120] Smith E. R. Semin G. R. (2004). Socially situated cognition: cognition in its social context. Adv. Exp. Soc. Psychol. 36, 53–117. 10.1016/S0065-2601(04)36002-8

[B121] Solarz A. K. (1960). Latency of instrumental responses as a function of compatibility with the meaning of eliciting verbal signs. J. Exp. Psychol. 59, 239–245. 10.1037/h0047274 13832584

[B122] Solomon K. O. Barsalou L. W. (2004). Perceptual simulation in property verification. Mem. Cogn. 32, 244–259. 10.3758/BF03196856 15190717

[B123] Sommerville J. A. Woodward A. L. Needham A. (2005). Action experience alters 3-month-old infants’ perception of others’ actions. Cognition 96, B1–B11. 10.1016/j.cognition.2004.07.004 15833301 PMC3908452

[B124] Stanfield R. Zwaan R. (2001). The effect of implied orientation derived from verbal context on picture recognition. Psychol. Sci. 12, 153–156. 10.1111/1467-9280.00326 11340925

[B125] Strack F. Deutsch R. (2004). Reflective and impulsive determinants of social behavior. Pers. Soc. Psychol. Rev. 8, 220–247. 10.1207/s15327957pspr0803_1 15454347

[B126] Strack F. Martin L. Stepper S. (1988). Inhibiting and facilitating conditions of the human smile: a nonobtrusive test of the facial feedback hypothesis. J. Pers. Soc. Psychol. 54, 768–777. 10.1037/0022-3514.54.5.768 3379579

[B127] Topolinski S. (2010). Moving the eye of the beholder: motor components in vision determine aesthetic preference. Psychol. Sci. 21, 1220–1224. 10.1177/0956797610378308 20639401

[B128] Topolinski S. Strack F. (2009). Motormouth: mere exposure depends on stimulus-specific motor simulations. J. Exp. Psychol. Learn. Mem. Cogn. 35, 423–433. 10.1037/a0014504 19271856

[B129] Tucker M. Ellis R. (1998). On the relations between seen objects and components of potential actions. J. Exp. Psychol. Hum. Percept. Perform. 24, 830–846. 10.1037/0096-1523.24.3.830 9627419

[B130] Urgesi C. Candidi M. Ionta S. Aglioti S. M. (2007). Representation of body identity and body actions in extrastriate body area and ventral premotor cortex. Nat. Neurosci. 10, 30–31. 10.1038/nn1815 17159990

[B131] Wheeler S. C. DeMarree K. G. Petty R. E. (2007). Understanding the role of the self in prime-to-behavior effects: the active-self account. Pers. Soc. Psychol. Rev. 11, 234–261. 10.1177/1088868307302223 18453463

[B132] Willems R. M. Casasanto D. (2011). Flexibility in embodied language understanding. Front. Psychol. 2:116. 10.3389/fpsyg.2011.00116 21779264 PMC3132681

[B133] Willems R. M. Francken J. C. (2012). Embodied cognition: taking the next step. Front. Psychol. 3:582. 10.3389/fpsyg.2012.00582 23293620 PMC3531608

[B134] Williams L. Huang J. Bargh J. (2009). The scaffolded mind: higher mental processes are grounded in early experience of the physical world. Eur. J. Soc. Psychol. 39, 1257–1267. 10.1002/ejsp.665 20046813 PMC2799930

[B135] Wilson M. (2002). Six views of embodied cognition. Psychon. Bull. Rev. 9, 625–636. 10.3758/BF03196322 12613670

[B136] Wollmer M. A. de Boer C. Kalak N. Beck J. Götz T. Schmidt T. (2012). Facing depression with botulinum toxin: a randomized controlled trial. J. Psychiatr. Res. 46, 574–581. 10.1016/j.jpsychires.2012.01.027 22364892

[B137] Xu A. J. Zwick R. Schwarz N. (2012). Washing away your (good or bad) luck: physical cleansing affects risk-taking behavior. J. Exp. Psychol. Gen. 141, 26–30. 10.1037/a0023997 21707206

[B138] Yan Z. Ding D. Yan L. (2011). To wash your body, or purify your soul: physical cleansing would strengthen the sense of high moral character. Psychology 2, 992–997. 10.4236/psych.2011.29149

[B139] Zajonc R. Markus H. (1988). “Affect and cognition: the hard interface,” in Emotions, Cognition, and Behavior, eds Izard C. E. Kagan J. Zajonc R. B. (Cambridge, UK: Cambridge University Press), 73–102.

[B140] Zhang M. Li X. (2012). From physical weight to psychological significance: the contribution of semantic activations. J. Consum. Res. 38, 1063–1075. 10.1086/661768

[B141] Zhong C. Liljenquist K. (2006). Washing away your sins: threatened morality and physical cleansing. Science 313, 1451–1452. 10.1126/science.1130726 16960010

[B142] Zwaan R. A. Taylor L. J. (2006). Seeing, acting, understanding: motor resonance in language comprehension. J. Exp. Psychol. Gen. 135, 1–11. 10.1037/0096-3445.135.1.1 16478313

[B143] Zwickel J. Prinz W. (2012). Assimilation and contrast: the two sides of specific interference between action and perception. Psychol. Res. 76, 171–182. 10.1007/s00426-011-0338-3 21556808

